# Brown Adipose Tissue and Metabolic Markers Differ Between Greenlanders and Danes With Cold Activation

**DOI:** 10.1210/clinem/dgaf615

**Published:** 2025-11-17

**Authors:** Mette Motzfeldt Jensen, Charlotte Elberling Almasi, Benedict Kjærgaard, Bodil Steen Rasmussen, Mette Korre Andersen, Mikkel Schubert, Torben Hansen, Christina Ellervik, Camilla Schéele, Marit Eika Jørgensen, Stig Andersen

**Affiliations:** Department of Clinical Medicine, Aalborg University, Aalborg 9000, Denmark; Greenland Center for Health Research, Ilisimatusarfik, University of Greenland, Nuuk 3900, Greenland; Department of Clinical Medicine, Aalborg University, Aalborg 9000, Denmark; Department of Nuclear Medicine, Aalborg University Hospital, Aalborg 9000, Denmark; Department of Clinical Medicine, Aalborg University, Aalborg 9000, Denmark; Biomedical Research Laboratory, Aalborg University Hospital, Aalborg 9000, Denmark; Department of Clinical Medicine, Aalborg University, Aalborg 9000, Denmark; Department of Anesthesia and Intensive Care, Aalborg University Hospital, Aalborg 9000, Denmark; Novo Nordisk Foundation Center for Basic Metabolic Research, University of Copenhagen, Copenhagen 2200, Denmark; Novo Nordisk Foundation Center for Basic Metabolic Research, University of Copenhagen, Copenhagen 2200, Denmark; Novo Nordisk Foundation Center for Basic Metabolic Research, University of Copenhagen, Copenhagen 2200, Denmark; Department of Clinical Biochemistry, Zealand University Hospital, Aalborg 9000, Denmark; Department of Clinical Medicine, Faculty of Health and Medical Sciences, University of Copenhagen, Copenhagen 2200, Denmark; Department of Laboratory Medicine, Boston Children's Hospital, Harvard Medical School, Boston, MA 02115, USA; Novo Nordisk Foundation Center for Basic Metabolic Research, University of Copenhagen, Copenhagen 2200, Denmark; Greenland Center for Health Research, Ilisimatusarfik, University of Greenland, Nuuk 3900, Greenland; Steno Diabetes Center Greenland, Queen Ingrids Hospital, Nuuk 3900, Greenland; Department of Clinical Medicine, Aalborg University, Aalborg 9000, Denmark; Greenland Center for Health Research, Ilisimatusarfik, University of Greenland, Nuuk 3900, Greenland; Department of Geriatric Medicine, Aalborg University Hospital, Aalborg 9000, Denmark

**Keywords:** brown adipose tissue, PET/CT scans, metabolism, lipids

## Abstract

**Context:**

Brown adipose tissue (BAT) is a thermogenic tissue that converts energy into heat in response to cold exposure, and is therefore a promising target for improving cardiometabolic health. Habitually cold-exposed Arctic people represent a unique model of metabolic adaptation to cold.

**Objective:**

We investigated cold-induced BAT activation in Greenlanders in comparison to Danes.

**Methods:**

A comparative crossover study was conducted of 13 Greenlanders and 11 Danes, with 2 experimental sessions conducted 2 weeks apart: A, a thermoneutral session; and B, a cooling session. BAT volume and activity were assessed using 2-deoxy-2-[^18^F]-fluoro-D-glucose ([^18^F]FDG) positron emission tomography/computed tomography (PET/CT) imaging. Serial measurement of metabolic markers associated with BAT activity was performed by blood sampling.

**Results:**

Both for Greenlanders and Danes, core temperature decreased 0.5 °C during cooling, and the volume of active BAT increased, yet with a higher increase in active BAT volume of 16 917% in Greenlanders compared to 4541% in Danes (*P* = .046). Marked differences in glucose levels were seen in Greenlanders and Danes in response to cooling (Danes: 0.30 mmol/L, *P* = .013; Greenlanders: 0.16 mmol/L, *P* = .12), insulin (Danes: 2.43 pmol/L, *P* = .32; Greenlanders: 6.65 pmol/L, *P* = .001), triglycerides (Danes: −0.59 mmol/L, *P* < .001; Greenlanders: −0.16 pmol/L, *P* = .21), and total cholesterol (Danes: 0.39 mmol/L, *P* < .001; Greenlanders: 0.02 mmol/L, *P* = .89).

**Conclusion:**

The larger increase in BAT activity in response to cooling and the dampened response in metabolic parameters in Greenlanders compared to Danes leads to a hypothesis of preparedness for rapid thermogenic response to cold exposure in populations with active BAT.

Brown adipose tissue (BAT) is a metabolically active thermogenic tissue primarily responsible for nonshivering thermogenesis, a physiological process whereby energy is dissipated as heat in response to cold exposure. Unlike white adipose tissue (WAT), which stores energy, BAT contains abundant mitochondria expressing uncoupling protein 1 (UCP1), enabling heat production via the proton leak pathway of oxidative phosphorylation (1).

BAT has definitively been identified in adult humans through positron emission tomography/computed tomography (PET/CT) imaging, which demonstrates metabolically active fat depots responsive to cold exposure (2-4). Since then, the presence of BAT has been associated with favorable metabolic outcomes, including lower body mass index (BMI), higher insulin sensitivity, and a lower risk of type 2 diabetes, dyslipidemia, and cardiovascular disease (5-7).

Cold environments strongly stimulate BAT activity by promoting cellular proliferation, upregulation of UCP1, and browning of white adipocytes, a process in which certain white fat cells acquire increased mitochondrial content and thermogenic capacity through UCP1 expression (8, 9). These so-called “beige” adipocytes are functionally similar to classic BAT cells. BAT oxidizes glucose and lipids to generate heat, effectively functioning as a biological heater. Hence, populations in need of heat production may be a target for the study of BAT activity. The Greenlandic Inuit represent such a population particularly relevant for studying BAT-related cold adaptation and metabolism, given their long-term residence and adaptation to the Arctic environment characterized by extreme cold and high dietary intake of fat. Genomic studies have identified several adaptive alleles in this population, particularly in genes involved in lipid metabolism such as *FADS* and *CPT1A*, which are believed to facilitate efficient utilization of lipid-rich diets typical of traditional subsistence lifestyles (10-12). These genetic adaptations and distinctive lipid profiles suggest a physiological framework optimized for thermogenesis and energy expenditure under chronic cold exposure. Notably, the Greenlandic Inuit exhibit a body composition characterized by shorter stature, higher BMI, and greater subcutaneous WAT deposition compared to White individuals. This Inuit phenotype is often accompanied by favorable metabolic profiles, suggesting a uniquely adapted adipose tissue function (13-15).

While BAT is increasingly recognized as an important site of energy expenditure and metabolic regulation in adult humans, most studies have focused on individuals of European ancestry, with limited inclusion of other ethnic groups. Indigenous Arctic populations, despite their longstanding exposure to extreme cold and their high relevance for understanding human cold adaptation, have received limited attention in BAT research (16).

This work aims to provide insight into the role of BAT in response to habitual and acute cold exposure by examining Greenlanders and matched Danes under controlled thermal conditions using 2-deoxy-2-[^18^F]-fluoro-D-glucose ([^18^F]FDG) PET/CT imaging. To complement the imaging data, we also assess circulating metabolic biomarkers to characterize the systemic physiological responses associated with BAT activation.

## Materials and Methods

### Design

This study employed a crossover comparative design involving Greenlanders and Danish participants. Each participant completed 2 trial sessions: A, a control session conducted under thermoneutral conditions, and B, a cooling session with 2 hours of individualized cooling to activate BAT. Blood samplings at defined time points were obtained, and both sessions concluded with a [^18^F]FDG PET/CT scan. The order of sessions was scheduled in a pseudo-random sequence based on logistical feasibility.

Participants were instructed to fast for 6 hours prior to each session and to refrain from smoking during this period. Additionally, they were advised to avoid extreme temperatures, vigorous physical activity, and high-fat meals in the 24 hours preceding each session.

All individuals underwent a screening process prior to final inclusion. The study was preregistered at ClinicalTrials.gov (identifier: NCT05884177; registered June 1, 2023).

Informed written consent was obtained from all participants prior to their inclusion in the study in accordance with the Declaration of Helsinki. The ethical board of North Denmark Region approved the experimental protocol (N-20220042).

#### Setting

This study was conducted at Aalborg University Hospital, Denmark, between September 18, 2023, and March 8, 2024.

### Inclusion

A total of 24 participants were enrolled, including 13 Greenlanders and 11 Danes, matched by age, sex, and BMI. Participants were recruited through social media and the Greenlandic House in Aalborg, Denmark. Interested individuals contacted the first author and were subsequently invited to an information meeting and screening visit. The participants’ ethnicity was self-reported and additionally characterized by grandparental origin and was later verified by genetic analyses. All Greenlandic participants had at least 2 grandparents born in Greenland.

**Figure 1.**
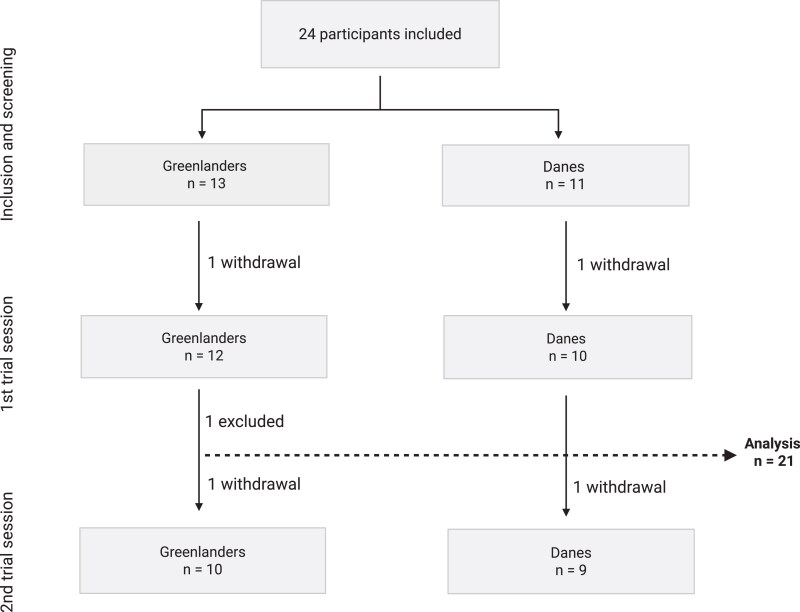
A flow diagram of participant inclusion and exclusion. Two participants concluded only their first trial session and are included in formal analysis. They were both missing session A with thermoneutral control scan and blood sampling. One participant was excluded after the first trial session due to incidental finding, and was not included in the formal analysis.

Fig. 1 illustrates the flow of participant inclusion. Two individuals withdrew after the screening process and did not attend any trial session. One participant was excluded following session 1 due to incidental findings on the PET/CT scan. All remaining participants completed the cooling session, while 2 did not complete the thermoneutral session. Consequently, data from 21 participants (11 Greenlanders and 10 Danes) were included in the analysis of the cooling session, and 19 participants (10 Greenlanders and 9 Danes) were included in the thermoneutral session analysis. Created in BioRender. Motzfeldt Jensen, M. (2025) https://BioRender.com.

#### Eligibility criteria

Methods are described in detail in a separate report (17). Participants were eligible if they were aged 18 to 50 years and of Danish or Greenlandic descent, defined by place of birth across 3 generations. Exclusion criteria included pregnancy, BMI greater than 35, random plasma glucose greater than 11 mmol/L, history of diabetes, thyroid or kidney disorders, recent cancer (within 3 years), prior obesity treatment, and substance or alcohol abuse (within 4 years). Use of medications affecting sympathetic nervous activity and regular winter swimming also led to exclusion. Known carriers of maturity-onset diabetes of youth or the TBC1D4 p.Arg684Ter variant, both associated with diabetes risk in Greenlandic Inuit, were excluded post hoc based on genetic screening.

#### Screening procedure

Prior to the trial sessions, all participants underwent a health screening to ensure eligibility. Anthropometric measurements included height, weight, BMI, waist, and hip circumference. Body composition was assessed using dual-energy x-ray absorptiometry (DXA). Blood samples were collected after an overnight fasting. Glucose, creatinine, hepatic enzyme levels (including alanine transaminase, aspartate transaminase, and alkaline phosphatase), C-reactive protein, and thyrotropin were within the reference ranges as required for inclusion. In addition, a 2-hour oral glucose tolerance test was performed to assess glucose metabolism. Participants were asked about current medication use, and medication records were verified through the national electronic patient journal system to assess eligibility.

### Cooling Intervention

During the cooling session, participants rested in a semi-supine position on a hospital bed, wearing standard hospital clothes. They were covered anteriorly and posteriorly over the truncus and legs with water-perfused cooling blankets connected to a temperature-regulated water-cooling system (Blanketrol III Hyper-Hypothermia System, Gentherm USA).

As recommended by BARCIST 1 guidelines, an individualized cooling protocol approach was followed (18). The duration of cooling was 120 minutes. Cooling exposure was individualized using a participant-guided approach based on thermal sensation. Blanket temperatures were continuously adjusted to maintain a subjective cold sensation rating of 8 to 9 on a visual analog scale, where 0 represented “very hot” and 10 indicated “very cold, cold shivering.” The aim was to achieve a cold sensation while avoiding muscle shivering. Shivering was monitored both subjectively and objectively using a handheld surface electromyography device (Clavis, Natus Neuro), which provided real-time auditory feedback in response to muscle contractions, allowing for early detection of involuntary muscle activation. Peripheral skin temperatures at the hands and feet were measured continuously using iButtons (Maxim Integrated Products) and recorded every 15 minutes. Core body temperature was continuously monitored via a rectal probe connected to Propaq MD (ZOLL Medical Corporation). This device also facilitated a precooling 3-lead electrocardiogram to confirm cardiovascular stability prior to initiating and during the 2-hour cooling protocol. Room temperature was kept stable during the cooling and monitoring of the participants.

The cold exposure did not precipitate any adverse events, and the cooling protocol was followed according to plan for all participants.

### Positron Emission Tomography/Computed Tomography Imaging

[^18^F]FDG PET/CT scans were conducted by certified personnel from the Department of Nuclear Medicine at Aalborg University Hospital, Denmark. A standard dose of 100-MBq [^18^F]FDG was administered via intravenous injection 60 minutes prior to image acquisition, in accordance with established protocols for BAT imaging (18).

### Positron Emission Tomography/Computed Tomography Analysis

Image analysis was performed using the PET/CT viewer plugin within the Fuji system. BAT was quantified using standardized uptake values (SUV) normalized for lean body mass (SUL), calculated as SUL = SUV/lean body mass. Lean body mass was determined from DXA-derived body composition data. An individualized threshold for BAT activity was applied, with regions considered positive for BAT if the SUL was 1.2 or greater and the corresponding tissue showed fat-like attenuation on CT (between −190 and −10 Hounsfield units). These criteria were based on established methodologies (18, 19).

Regions of interest were manually delineated in anatomically relevant areas known to harbor BAT, including the bilateral supraclavicular, cervical, axillary, paravertebral, and perirenal regions. These regions of interest were collectively analyzed to derive a total BAT measure, reflecting the overall volume of active BAT and metabolic activity of BAT in each participant. In addition to this composite analysis, a subanalysis was performed specifically for abdominal BAT to explore potential regional differences in BAT activity between participant groups. Volumetric and metabolic parameters, BAT volume (mL), SUVmean, and SUVmax and SUVpeak, were extracted for each participant.

### Blood Sampling and Biochemical Analysis

During the cooling session, venous blood samples were collected from a central venous catheter placed in the femoral vein at −15, 0, 30, 60, 90, 115, and 180 minutes. During the thermal comfort session, samples were obtained at −15, 0, 30, 60, and 115 minutes from a peripheral venous catheter inserted in the antecubital vein.

Blood samples were spun, and plasma or serum aliquots were stored at −80 °C until analysis. All biochemical measurements were performed at the Department of Clinical Biochemistry at Aalborg University Hospital (Aalborg, Denmark) after completion of all trials. Plasma concentrations of glucose, total cholesterol, high-density lipoprotein (HDL) cholesterol, and triglycerides were analyzed using the Alinity c-series analyzer (Abbott Laboratories). Detection limits and reference intervals according to the manufacturer were as follows:

- Plasma glucose: detection limit 0.28 mmol/L; reference interval 4.1-5.6 mmol/L
- Total cholesterol: detection limit 0.13 mmol/L; reference value less than 5.18 mmol/L
- HDL cholesterol: detection limit 0.13 mmol/L; reference value greater than 1.55 mmol/L
- Triglycerides: detection limit 0.06 mmol/L; reference value less than 1.70 mmol/L

Low-density lipoprotein (LDL) cholesterol was calculated using the Friedewald equation: LDL cholesterol = total cholesterol—HDL cholesterol—(triglycerides/2.2), where all concentrations are expressed in mmol/L. This method is valid only when triglyceride levels are below 4.5 mmol/L; no participants in this study exceeded this threshold.

Serum insulin concentrations were measured using an electrochemiluminescence immunoassay (Elecsys, Roche Diagnostics) on a Cobas 6000 analyzer. The assay had a detection limit of 1.39 pmol/L, with reference intervals ranging from 17.8 to 173 pmol/L. To minimize between-assay variability, all samples from the same participant were analyzed within the same run.

All participants were whole-genome sequenced with an average depth of 30× using the MGISEQ-T7 platform. Sequencing data were processed, mapped, and genotyped following the procedure previously described (20), using PALEOMIX rev. 1d5b491. Variant annotation was performed along with 371 unpublished Greenlandic samples with whole-genome sequence information. Based on the resulting genotype data, Inuit and European admixture proportions were calculated using ADMIXTURE v1.3.0 (21), using chip-genotyping data from a panel of 5996 Greenlanders as background (22). These calculations were based on a subset of biallelic variants selected using Plink v1.9.0-beta7.7 (23), with minor allele frequency greater than 5%, genotype missingness less than 1%, individual missingness less than 10%, and linkage-disequilibrium pruned within 1 Mb removing variants with *R^2^* greater than 0.8.

### Sample Size

BAT has not previously been measured in Inuit populations using [^18^F]FDG-PET/CT scans. Hence, we based our calculation on another study of BAT (24) and used a variation of 50% and a BAT volume of 130 compared to 330 mL in ethnic Danes and Greenlandic Inuit. Our estimates are based on an expected difference in BAT mass similar to the largest differences seen in the previous study (24). Using a significance level of 5% and a statistical power of 80%, we calculated that n = 8 participants are required in each group. We thus aimed for 10 participants in each group (17).

### Statistical Methods

All statistical analyses were performed using Stata version 18 (StataCorp LLC). A *P* value less than .05 was considered statistically significant.

Descriptive statistics were computed for all variables. Continuous variables are reported as means and SDs unless otherwise noted. Data distribution was visually evaluated using histograms and Q-Q plots.

Mixed-effects linear regression models were used to evaluate the effect of cooling (vs thermal comfort) on biomarkers and BAT parameters, and whether responses differed by ethnic group. Models included session, ethnic group, their interaction, and a carryover term, with age, sex, and BMI as covariates. A random intercept for participant accounted for repeated measures.

For selected biomarkers, time point–specific effects were explored, and an additional analysis assessed overall changes across all time points during the cooling session.

Assumptions of normality were assessed for key model residuals. Due to skewed distributions, BAT outcomes were log-transformed prior to modeling. Model estimates were back-transformed and expressed as relative (%) changes. Group differences in BAT response to cold were further assessed using robust linear regression on the within-participant change (Δlog(BAT)). Within the Greenlandic group, associations between the proportion of Inuit genetic admixture and BAT responsiveness were examined using robust linear regression, with admixture included as a continuous predictor and the within-participant log-transformed change in BAT outcomes as the dependent variable. Models were adjusted for age, sex, and BMI.

To summarize the integrated metabolic responses during each session, incremental areas under the curve (iAUCs) were calculated for all biomarkers. Baseline was defined as the mean of values obtained at −15 and 0 minutes. Baseline-adjusted concentrations were integrated from 0 to 115 minutes using the trapezoidal rule. Group- and session-specific mean ± SE values were summarized descriptively to illustrate the magnitude and direction of metabolic responses.

## Results

We observed no statistically significant differences in baseline characteristics between Greenlanders and Danes (Table 1). The genetic ancestry was 99% European for all Danish participants, while the Greenlanders had a mean Inuit ancestry of 50% (range, 27%-66%).

**Table 1.** Baseline characteristics of the study participants

	Danes	Greenlanders	Total
n (%)	10 (47.6)	11 (52.4)	21 (100.0)
Sex, n (%)			
Female	6 (46.2)	7 (53.8)	13 (100.0)
Male	4 (50.0)	4 (50.0)	8 (100.0)
Age, y	34.6 (8.3)	35.1 (8.4)	34.9 (8.1)
Height	174.7 (8.4)	169.8 (9.3)	172.1 (9.0)
Weight	77.4 (5.4)	77.1 (13.8)	77.2 (10.4)
BMI	25.5 (2.8)	26.8 (4.3)	26.1 (3.6)
Waist circumference, cm	79.8 (4.3)	86.9 (15.3)	83.5 (11.7)
Hip circumference, cm	99.5 (14.4)	102.4 (9.0)	101.0 (11.7)
Lean body mass, kg	52.6 (6.6)	51.3 (8.3)	51.9 (7.4)
Body fat, %	31.9 (8.7)	33.7 (7.0)	32.9 (7.7)
Hemoglobin A_1c_, mmol/mol	32.4 (1.3)	33.8 (2.4)	33.1 (2.1)
HOMA-IR	1.0 (0.6)	1.8 (1.2)	1.4 (1.0)

Baseline characteristics of participants stratified by ethnic group. Data are presented as mean ± SD for continuous variables, and n (%) for categorical variables, and all had *P* greater than .1.

Abbreviations: BMI, body mass index; HOMA-IR, homeostatic model assessment of insulin resistance.

**Figure 2.**
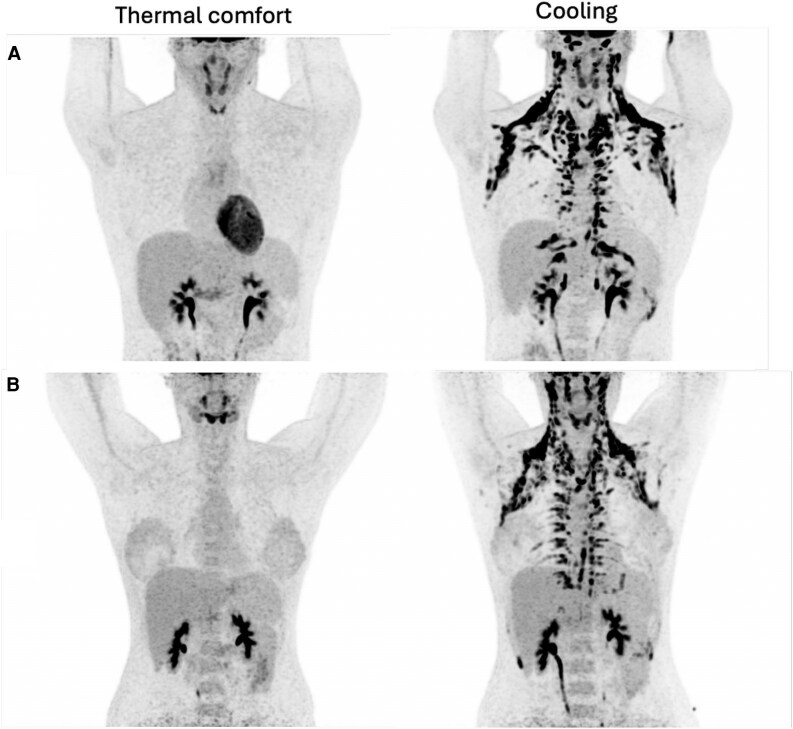
Positron emission tomography images of A, 1 Greenlander and B, 1 Danish participant at thermal comfort and following 2 hours of cooling.

There was no statistically significant difference in core temperature between groups at baseline (*P* = .48), and both groups exhibited a gradual and similar linear decline of 0.5 °C (95% CI, 0.31-0.74) after 2 hours cooling (Fig. 2). Both groups also exhibited a decrease in skin temperature after 2 hours cooling for upper (Danes: −2.7 °C, *P* < .01; Greenlanders: −2.1 °C, *P* < .01) and lower extremities (Danes: −4.8 °C, *P* < .001; Greenlanders: −4.3 °C, *P* < .001). Compared to Danes, Greenlanders had higher skin temperatures before cooling and maintained higher temperatures throughout the cooling: 1.1 °C higher in both lower (*P* = .039) and upper extremities (*P* = .12) (Supplementary Fig. S1) (25).

**Figure 3.**
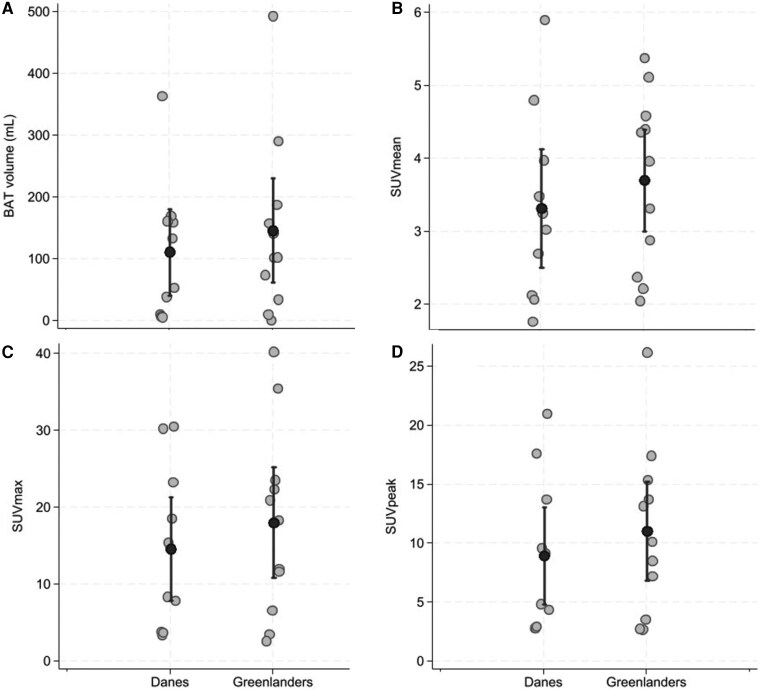
Cold-activated brown adipose tissue (BAT) in Greenlanders and Danes. Individual values of volume of A, active BAT; and B, mean; C, maximum; and D, peak standard uptake values (SUVs) in Danes and Greenlanders. Each dot represents one participant, and the black dots indicate group means with vertical black bars representing 95% CIs.

Table 2 shows the predicted geometric means and 95% CIs for BAT volume and activity (SUVmean, SUVmax, and SUVpeak) at thermal comfort and cooling adjusted for age, sex, and BMI, and the percentage increase. The increase with cooling was marked for all parameters except SUV mean among Danes, and the increase was 2 to 7 times higher among Greenlanders compared to Danes. The increase in BAT with cooling is illustrated for each participant in the Greenlandic and Danish group with group means and CIs in Fig. 3.

**Table 2.** Predicted geometric means (95% CI) and percentage increase of brown adipose tissue volume and activity

	Group	TC	Cooling	% increase	*P*, cooling vs TC
BAT volume, mL					
	Danes	1.36 (−0.04 to 2.8)	61.83 (1.6 to 122.0)	4541%	<.001
	Greenlanders	0.50 (0.005 to 1.0)	84.22 (5.8 to 162.7)	16 917%	<.001
SUVmean					
	Danes	2.19 (0.65 to 3.72)	3.45 (1.15 to 5.74)	58%	.34
	Greenlanders	0.71 (0.25 to 1.16)	2.58 (1.36 to 5.79)	408%	<.001
SUVmax					
	Danes	2.90 (0.96 to 4.84)	11.41 (4.20 to 18.62)	294%	.003
	Greenlanders	1.66 (0.61 to 2.70)	13.71 (0.61 to 2.70)	728%	<.001
SUVpeak					
	Danes	2.49 (0.94 to 4.03)	7.36 (3.04 to 11.67)	196%	.011
	Greenlanders	1.37 (0.57 to 2.16)	8.82 (3.97 to 13.67)	544%	<.001

Estimates are from mixed-effects regression models of log-transformed BAT-outcomes, adjusted for age, sex, and BMI. Values are back-transformed from the log-scale.

Abbreviations: BAT, brown adipose tissue; SUV, standard uptake value; SUV max, maximum standard uptake values; SUV peak, peak standard uptake values; TC, thermal cooling.

The log-transformed increase in volume of active BAT was significantly greater in Greenlanders (β = 1.76; 95% CI, 0.04-3.48; *P* = .046), corresponding to a 5.8-fold higher cold-induced BAT response compared to Danes. Greenlanders demonstrated a 4-fold greater SUVmean response to cooling compared to Danes (*P* = .063) with similar findings for SUVmax (*P* = .069) and SUVpeak (*P* = .075) (Supplementary Table S1) (25). Furthermore, statistical modeling using robust linear regression within the Greenlandic group showed that a higher proportion of Inuit genetic admixture was associated with a slight decrease in log-transformed BAT volume (β = −6.6 × 10⁻⁶; 95% CI, −9.9 × 10⁻⁶ to −3.2 × 10⁻⁶; *P* = .004) and SUVmean (β = −1.2 × 10⁻⁵; 95% CI, −1.9 × 10⁻⁵ to −5.2 × 10⁻⁶; *P* = .006). No associations were observed between Inuit genetic admixture and SUVmax or SUVpeak among Greenlanders (Supplementary Table S2) (25).

Abdominal BAT, defined as active BAT volume below the diaphragm exceeding 1 mL, was detected in 5 of 10 Danes and 4 of 11 Greenlanders. When including all participants, the median abdominal BAT volume was 0.96 mL (interquartile range [IQR]: 0.00-4.34 mL) in Danes and 0.62 mL (IQR: 0.00-4.17 mL) in Greenlanders. One Greenlandic participant exhibited a markedly higher volume of 34 mL. Among participants with detectable abdominal BAT, the median volume was 4.34 mL (IQR: 1.41-4.87 mL) in Danes and 6.98 mL (IQR: 4.09-22.00 mL) in Greenlanders.

Fig. 2 displays PET images from a Greenlandic and a Danish participant, both demonstrating abdominal BAT.

**Figure 4.**
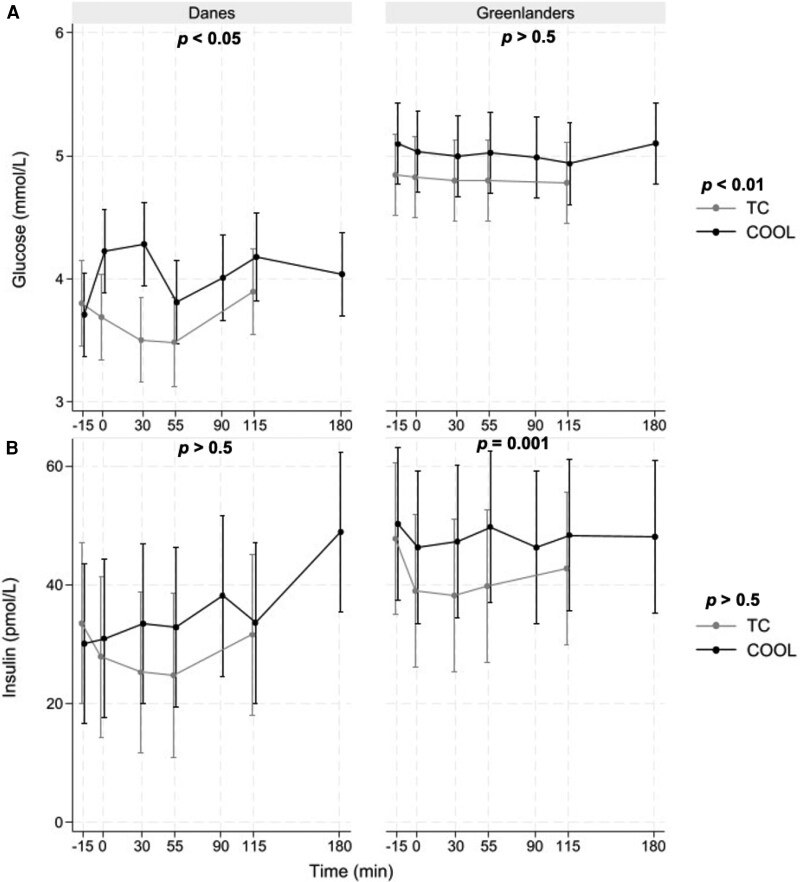
Time-concentration curves of A, glucose and B, insulin during thermal comfort (TC, gray) and cooling (COOL, black) sessions in Danes and Greenlanders. Lines represent model-estimated marginal means (±SE) derived from linear mixed-effects models. *P* values at the top of each panel indicate the effect of cooling compared with TC within each group, while *P* values on the right indicate between-group differences (Danes vs Greenlanders) during the thermal comfort session.

At baseline, Greenlanders had significantly higher levels of glucose than Danes (*P* < .001). In response to cooling, Danes exhibited a statistically significant increase in glucose levels (mean change: 0.30 mmol/L; *P* = .013), with an initial rise peaking before 55 minutes, followed by a near-normalization and a gradual secondary increase. In contrast, glucose concentrations in Greenlanders remained stable throughout the cooling period (Fig. 4). Although values tended to be slightly higher during cooling compared to thermal comfort, the difference was not statistically significant. Supplementary Fig. S2 (25) shows a box plot of glucose concentrations in Danes and Greenlanders during thermal comfort and cooling.

Cooling increased glucose iAUC in Danes (65.8 ± 28.8 mmol·min/L) compared with thermal comfort (−26.6 ± 23.0 mmol·min/L), whereas Greenlanders showed minimal change (−11.4 ± 5.0 vs −4.1 ± 3.1 mmol·min/L) (Supplementary Table S3) (25).

For insulin, there were no differences in levels between groups at baseline or post cooling. Insulin was higher in Greenlanders during the cooling session compared to thermal comfort (*P* = .001), with higher levels observed at time points 0 (7.75 mmol/L; *P* = .027), 30 (9.65 mmol/L; *P* = .006), and 55 minutes (10.20 mmol/L; *P* = .004). The Danish group showed an increase in insulin at a later time point, with a rise of 18.60 mmol/L after 2 hours of cold exposure (*P* < .001) (see Fig. 4).

**Figure 5.**
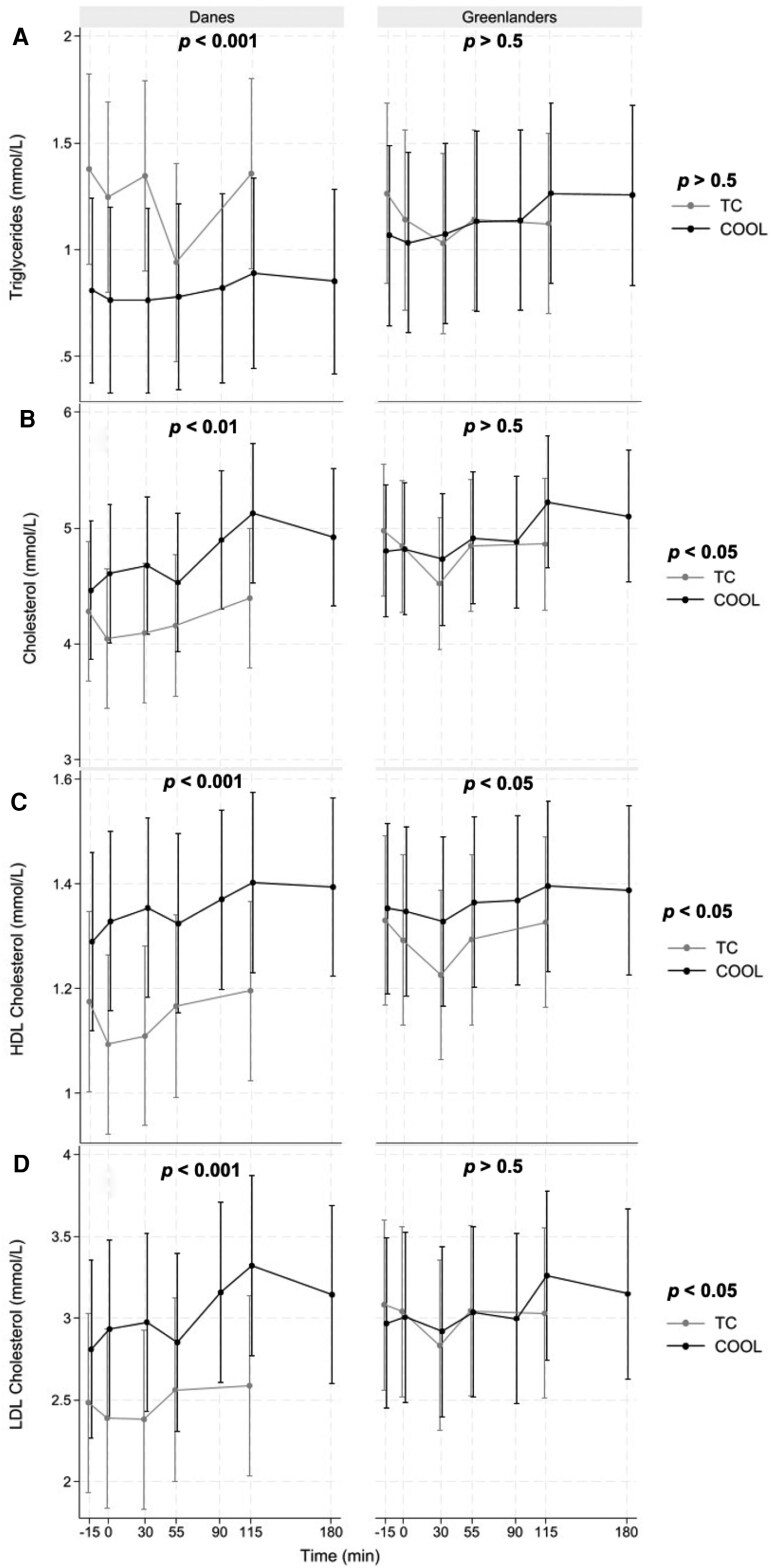
Time-concentration curves of A, triglycerides; B, total cholesterol; C, high-density lipoprotein (HDL); and D, low-density lipoprotein (LDL) during thermal comfort (TC, gray) and cooling (COOL, black) sessions in Danes and Greenlanders. Lines represent model-estimated marginal means (±SE) derived from linear mixed-effects models. *P* values in the top of each separate figure represent the effect of cooling compared to TC both for Danes and Greenlanders. The *P* values in the right side of the figures represent the difference at thermal comfort sessions for Danes compared to Greenlanders.

Fig. 5 illustrates lipid dynamics during the cooling and thermoneutral sessions. In the Danish group, triglyceride levels were significantly lower during cooling compared to thermoneutral conditions (*P* < .001), with differences observed at time points −15, 0, 30, and 115 minutes. However, triglyceride levels remained stable during cooling. In the Greenlandic group, triglyceride levels increased relative to baseline (−15 minutes) at time points 115 and 180 minutes (0.19 mmol/L, *P* < .001; 0.18 mmol/L, *P* = .001) during cooling, but did not differ between thermal comfort and cooling.

Among Danes, total cholesterol was on average 0.4 mmol/L higher during the cooling session compared to the thermoneutral condition (*P* = .003). An additional rise occurred toward the end of cooling (time point 115 minutes, *P* < .001; 180 minutes, *P* = .002). In Greenlanders, total cholesterol remained stable throughout most of the cooling session but was higher at 115 (0.40 mmol/L; *P* = .008) and 180 minutes (0.29 mmol/L; *P* = .050) compared to time point −15 minutes, but did not differ between thermal comfort and cooling. Total cholesterol iAUC increased during cooling in Danes (37.1 ± 32.3 mmol·min/L) but remained close to zero in Greenlanders (3.5 ± 9.5 mmol·min/L) (Supplementary Table S3) (25).

Cooling significantly increased HDL cholesterol in both groups (Danes: 0.20 mmol/L, *P* < .001; Greenlanders: 0.07 mmol/L, *P* = .01). Among Danes, HDL levels were higher at all time points during the cooling session, both at precooling time point −15 minutes (*P* = .007), and at all subsequent time points (*P* < .001). In contrast, Greenlanders showed a modest effect, with a statistically significant difference only at time point 30 minutes (*P* = .032). HDL cholesterol iAUC rose modestly with cooling in Danes (10.2 ± 8.1 mmol·min/L) but was unchanged in Greenlanders (−0.5 ± 2.9 mmol·min/L) (see Supplementary Table S3) (25).

LDL cholesterol was 0.47 mmol/L higher during the cooling session compared to thermoneutral among Danes (*P* < .001), with marked differences at time points −15, 0, 30, and 115 minutes. During the cooling session, LDL levels increased significantly at time points 115 (0.47 mmol/L; *P* < .001) and 180 minutes (0.34 mmol/L; *P* = .002) relative to baseline. In contrast, Greenlanders showed no overall change in LDL cholesterol in response to cold exposure (*P* = .87), apart from a modest increase at 115 minutes (0.27 mmol/L; *P* = .02) relative to baseline. LDL cholesterol iAUC increased with cooling in Danes (29.0 ± 25.1 mmol·min/L) and remained low in Greenlanders (1.8 ± 7.4 mmol·min/L) (see Supplementary Table S3) (25).

## Discussion

The cooling protocol induced a steady decline in core temperature by 0.5 °C, which successfully activated BAT in the majority of participants. Following cooling, the volume of active BAT increased around 45-fold in Danes and 170-fold in Greenlanders as evaluated from the [^18^F]FDG PET/CT scans.

Within the Greenlandic group, we explored whether the degree of Inuit genetic admixture was associated with BAT responsiveness. Contrary to our initial hypothesis that higher Inuit ancestry would predict greater BAT activation due to long-term cold adaptation, we observed a modest inverse association between admixture and BAT activation. The analysis was based on a small subsample, with modest heterogeneity in admixture levels (27%-66%), and the study was not designed to stratify individuals by genetic ancestry. Therefore, the observed inverse association of BAT activation and Inuit admixture should be interpreted with caution.

At the group level, Greenlanders exhibited greater activated BAT volume than Danes. This observation is consistent with previous studies suggesting that cold exposure during early development can enhance BAT function later in life. A recent study reported elevated BAT activity in individuals conceived during colder seasons (26). Similarly, Levy et al (27) demonstrated that early-life cold exposure, particularly during childhood, can lead to increased BAT activity in adulthood, supporting the hypothesis that developmental plasticity contributes to long-term thermogenic capacity.

Even though the Greenlandic participants had lived in Denmark for years, they were born in Greenland and therefore likely to have spent more time outdoors in Arctic environments relative to Danes. The higher BAT volume may reflect transgenerational or genetic effects.

Previous studies have demonstrated that the adipose tissue phenotype in Greenlanders differs from that of individuals of European descent. Specifically, Inuit tend to have abundant subcutaneous WAT, which, unlike in White individuals, is associated with a healthier metabolic profile (14). Studies at single-cell resolution of adipose tissue from White individuals have suggested that adipocytes with thermogenic signature are present in visceral but not subcutaneous abdominal adipose tissue (28, 29). While the persistence of beige adipocytes in human subcutaneous fat remains debated, seasonal variation in UCP1 expression has been reported in subcutaneous WAT, with higher levels during colder months (30). Additionally, thermogenic cells in WAT have been identified with functions extending beyond UCP1 expression (31, 32). Together, these observations raise the possibility that the subcutaneous WAT in Greenlanders may harbor thermogenic adipocytes. In mice the browning process was earlier studied in vivo using different imaging modalities, including CT, xenon-enhanced CT, dynamic contrast-enhanced ultrasound, and [^18^F]FDG-PET/CT (33). A small but significant increase in radiodensity indicative of either an increase in tissue vascularization and/or cell water and protein content, and a slight increase in xenon uptake indicative of an increase of perfusion, was found. However no statistically significant increase in glucose uptake was found using PET/CT. In the present study, we did not assess subcutaneous beige adipose tissue. Whether this is due to limited sensitivity inherent to the modality or lack of glucose uptake is unknown (33); however, we may have underestimated the difference in total thermogenic potential in these populations.

As for lipids, BAT contributes to lipid metabolism by facilitating the clearance of triglyceride-rich lipoproteins and promoting reverse cholesterol transport, which may improve circulating levels of triglycerides, HDL, and LDL cholesterol (34, 35). Cold exposure activates the sympathetic nervous system and increases catecholamine release, which stimulates lipolysis in WAT and mobilizes circulating fatty acids as substrates for thermogenesis. In parallel, activated BAT upregulates lipoprotein lipase and facilitates the uptake and oxidation of fatty acids derived from circulating triglyceride-rich lipoproteins.

During short-term cold exposure, BAT initially oxidizes intracellular triglyceride stores. Consequently, systemic changes in circulating lipid levels tend to emerge after prolonged or sustained BAT activation (36).

In the present study, lipid responses to cold exposure differed between Greenlanders and Danes. While Danes exhibited lower levels of triglycerides during thermal comfort and increased levels of total and LDL cholesterol during cooling, Greenlanders maintained stable lipid levels. A modest rise in HDL cholesterol was also observed in Greenlanders during cooling, though the overall lipid response showed markedly less dynamics compared to the Danish participants. Notably, in Danes, differences in lipid levels were already apparent at precooling time points (−15 and 0 minutes) during the cooling session compared to thermal comfort. This may reflect anticipatory physiological responses to the cold exposure, differences in metabolic state, or circadian variation between test days. Nonetheless, changes were also clearly observed during the cooling period itself, suggesting a true cold-induced effect.

These findings may reflect ethnic or physiological differences in BAT activation, lipid mobilization, or broader metabolic regulation in response to cold exposure. Hence, the lipid trajectories conform to a response in lipids in both groups beyond the 2 hours of cooling, suggesting that pronounced effects might have emerged with extended cold exposure. In a study with 5 hours of cold exposure, no acute changes in triglycerides or cholesterols were detected; however, next-day reductions in fasting triglycerides and very-LDL cholesterol were observed, indicating a delayed modulation of lipid metabolism (36). Thus, our findings indicate that short-term protocols may underestimate the full metabolic effect of BAT activation. Importantly, that study also reported increased plasma free fatty acids and glycerol concentrations (36), which were not assessed in the present work, but may likewise have revealed additional cold-induced effects.

Baseline homeostatic model assessment of insulin resistance levels indicate insulin-sensitive participants in both groups, with no statistically significant difference between groups. Cold exposure influences glucose homeostasis through multiple mechanisms (37). Systemic glucose concentrations reflect the net balance of peripheral uptake and release of glucose. Activated BAT increases peripheral glucose uptake and improves insulin sensitivity (38), yet cold exposure also increases glucagon and stimulates hepatic gluconeogenesis (39, 40). Danes exhibited an increase in circulating glucose during cooling, whereas Greenlanders maintained stable glucose levels. The increased glucose levels in Danes may reflect mobilization of substrates, with hepatic glucose output exceeding the rate of glucose uptake by BAT and other peripheral tissues. Conversely, the stable glucose levels in Greenlanders suggest differences in substrate utilization, potentially with a greater reliance on fatty acid oxidation. This metabolic profile may indicate a physiological preparedness to cold in Greenlanders, allowing them to maintain glucose homeostasis despite increased metabolic demands.

This pattern could mirror the dynamics observed for circulating triglycerides, which require prolonged cooling to influence the extracellular triglyceride concentrations. Hence, our data raise the hypothesis that in Greenlanders, BAT is primed for rapid thermogenic activation on cold exposure, including mechanisms independent of external fueling. Such mechanisms are consistent with the unique profile for metabolizing both glucose and lipids among Greenland Inuit (10-12). Dynamic PET scanning may provide evidence for such mechanisms of BAT preparedness and is a path to be pursued in further studies.

The findings of this study provide insights into the metabolic responses based on experimentally induced acute cold exposure; however, the study did not investigate the underlying cellular metabolic mechanisms. These may involve genetic factors or individual variation in environmental adaptation with sympathetic nervous system regulation, thyroid hormone activation, or differences in mitochondrial UCP1 expression. This may guide the design of future studies aimed at elucidating the physiological pathways involved.

### Strengths and Limitations

Measurement of the Inuit admixture proportion strengthens our analysis, particularly given that all Greenlandic participants were residing in Denmark during the study period. This relocation to a milder climate is likely to have influenced cold exposure and associated behavioral factors, such as time spent outdoors and dietary habits. While the lack of knowledge on the duration of residence at lower latitudes of Denmark is a limitation, it is possible that differences in cold-adaptive traits would have been more evident with a population with a higher degree of Inuit ancestry.

Individuals with reduced cold tolerance may have been less likely to volunteer for participation, potentially leading to underrepresentation of this subgroup. This potential bias was similar both for Greenlanders and Danes. However, as the Greenlandic participants were residents of Denmark, it is conceivable that they had a lower habitual cold exposure and, consequently, reduced cold tolerance compared with the general Greenlandic population. Such a bias would likely attenuate, rather than exaggerate, the observed differences between groups.

The crossover design is a strength with each participant being his or her own control. However, the order of experimental sessions was determined using a pseudo-randomized sequence, primarily guided by logistical feasibility, including the availability of cooling equipment and PET/CT scanner slots. While not randomized, this approach helped minimize systematic bias related to session timing, and findings did not differ by order of session supported by the 2-week interim between sessions. While blinding was not possible for cooling or ethnicity during data collection, laboratory analyses were conducted without knowledge of participant group or time point, and analysis for each participant was performed in the same run to prevent between-batch variation.

BAT uses both glucose and fatty acids as substrates for thermogenesis. This metabolic activity allows for the use of PET/CT imaging with the glucose analogue [^18^F]FDG as a well-established method to quantify BAT activity. Quantification of BAT parameters was performed using individualized SUV thresholds adjusted for lean body mass, derived from DXA scans, in accordance with instructions for BAT imaging and quantification (19). This methodological approach enhances the accuracy and physiological relevance of BAT assessment across participants with varying body compositions. However, it is important to recognize that [^18^F]FDG uptake is not exclusive to BAT and may reflect other metabolic processes. In our study, we found higher [^18^F]FDG uptake values in the Danish participants during thermal comfort. The elevated signal could indicate a greater degree of nonspecific uptake, potentially related to vascular inflammation or early stages of atherosclerosis. PET tracers specific for BAT detection are still needed (41, 42). Finally, one Greenlander was unaffected on PET by cooling despite the cooling equipment working at maximum capacity. This participant had a high waist circumference and a BMI of 32. However, excluding this participant did not alter results.

## Conclusion

Cooling increases the volume and intensity of active BAT both in Danish and Greenlandic adults. Interestingly, a higher amount of cold-induced BAT was observed among Greenlanders compared to Danes. Moreover, the metabolic effects during cooling differed between the two groups, suggesting a preparedness for rapid activation of BAT among the participants of Arctic descent. Such a difference in metabolic capacity could contribute to health effects among cold-habituated people and potentially unveil paths for interventions on cardiometabolic health and obesity.

## Data Availability

Some or all datasets generated during and/or analyzed during the current study are not publicly available but are available from the corresponding author on reasonable request.

## Clinical Trial Information

ClinicalTrials.gov trial registration number NCT05884177 (registered June 1, 2023).

## Abbreviations

[^18^F]FDG: 2-deoxy-2-[^18^F]-fluoro-D-glucose
BAT: brown adipose tissue
BMI: body mass index
CT: computed tomography
DXA: dual-energy x-ray absorptiometry
HDL: high-density lipoprotein
iAUCs: incremental areas under the curve
LDL: low-density lipoprotein
PET: positron emission tomography
SUL: standardized uptake values normalized for lean body mass
SUV: standard uptake value
SUVmax: maximum standard uptake values
SUVpeak: peak standard uptake values
UCP1: uncoupling protein 1
WAT: white adipose tissue
